# Supplementary material for: Proteomic Analysis of Plants with Binding Immunoglobulin Protein Overexpression Reveals Mechanisms Related to Defense Against Moniliophthora perniciosa
Source: Plants (Basel). 2025 Feb 7;14(4):503. doi: 10.3390/plants14040503 (PMC11859623; doi:10.3390/plants14040503)
Supplement: Supplementary file 1 [file plants-14-00503-s001.zip › plants-3427143-supplementary.pdf]

Supplementary Materials:

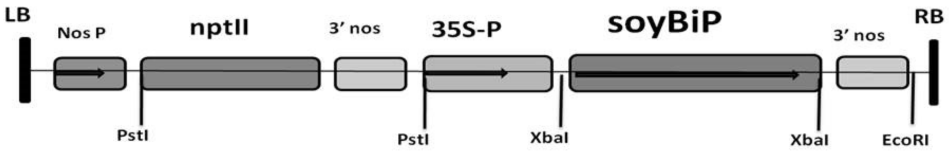

Figure S1: Construction used for genetic transformation of the Micro-Tom tomato variety. Vector pUFV42, the soyBiP gene in the sense direction under control of the constitutive promoter containing CAMV35S and the polyadenylation signal (3' nos). LB and RB correspond to the left and right edges of the T-DNA, respectively. Adapted from [32].

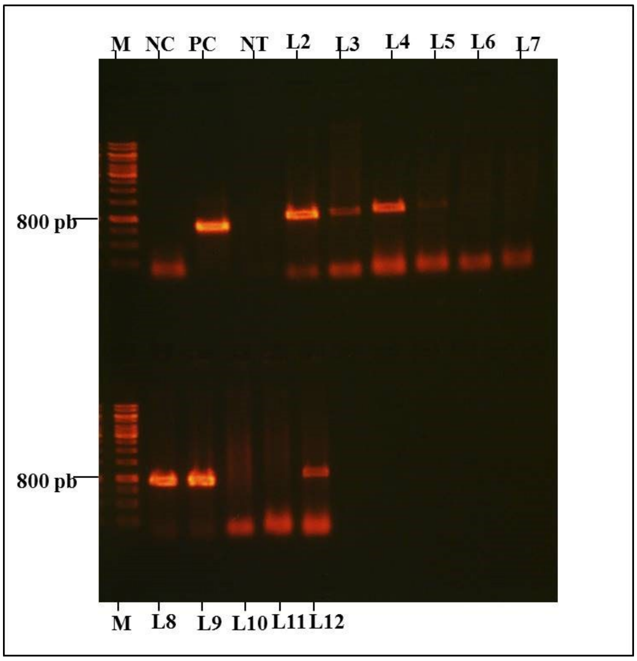

Figure S2: Molecular diagnosis of *S. lycopersicum* **plants expressing** ***Soy*****BiP*****D*****.** Genomic DNA subjected to PCR reaction using primers for *nptII* (Neomycin Phosphotransferase). **(M)**, 1 Kb marker **(CN)** Negative control - reaction without using template DNA, **(CP)** Positive control - reaction using pUFV42 vector as template, **(NT)** Non-transformed plant, **(L2 to L12)** lines regenerated in vitro. 800pb expected size of the fragment to be amplified.

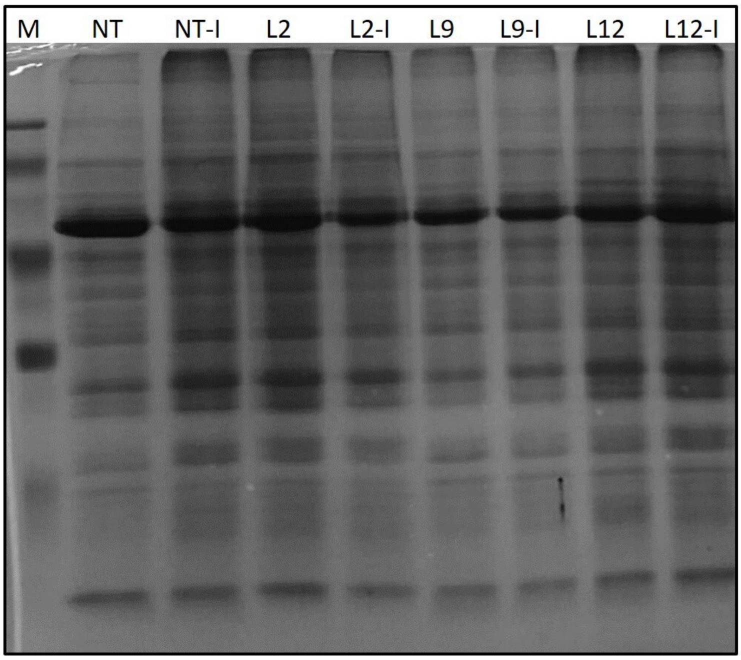

Figure S3: Protein analysis of transgenic plants. 40 µg of total proteins extracted: from non-transformed **(NT)** or transgenic **(L2 to L12)** ***S. lycopersicum*** leaves were resolved on polyacrylamide gel and visualized after staining with Comassie Blue. **M**: Low Molecular Marker GE Healthcare (97, 66, 45, 30, 20 KDa).

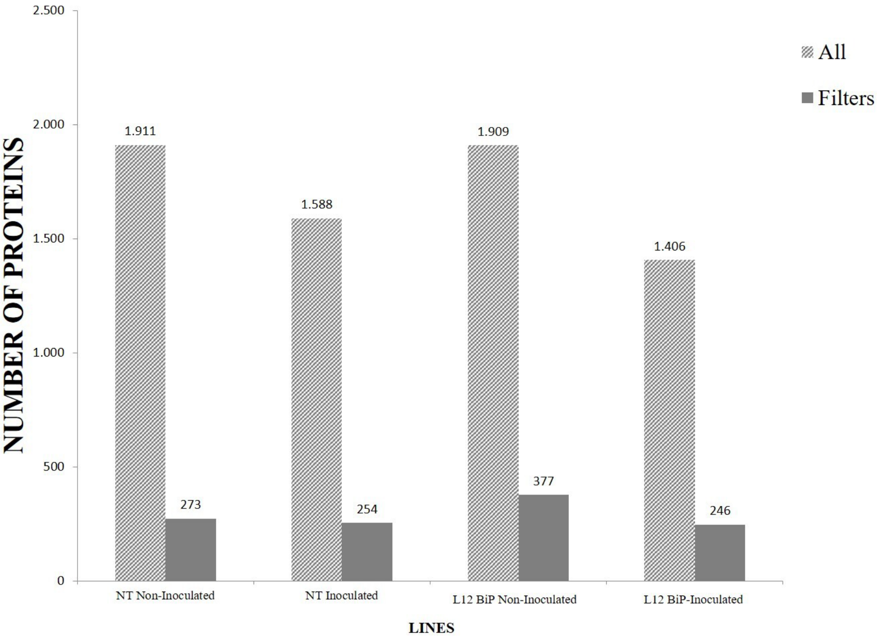

Figure S4. Total number of identified proteins and number of proteins after statistical filtering (based on identification in 100% of triplicates). **(NT)** non-transformed plant **(L12 BiP)** transgenic line.

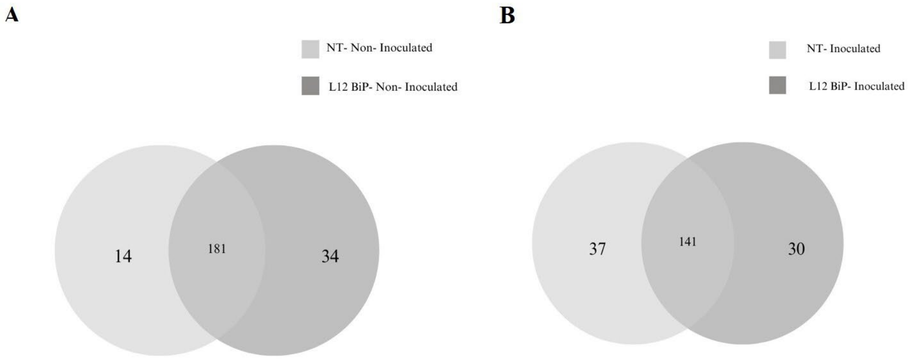

Figure S5. Venn diagrams. Distribution of unique and differentially abundant proteins identified among the lines**. (A)** NT treatment *versus* L12 BiP Non- inoculated. **(B)** NT treatment *versus* L12 BiP Inoculated with *M. perniciosa.* **(NT)** plant line Non-transformed **(L12 BiP)** plant line overexpressing BiP.

Table S1. Identification of unique and differential proteins NT *versus* L12 BiP treatment Non- inoculated. (NT): Non-transformed **plant; (L12 BiP)** Transgenic line.

Nº Acess	*Protein Name*	*Abundance L12 BiP*	*Abundance Non-transformed*	*Biologic Process*	*Aminoacids*
A0A3Q7HX02	luminal-binding protein precursor	11,163112	0	endoplasmic reticulum unfolded	673
A0A3Q7HDP5	Aspartic proteinase	5,3342648	0	proteolysis	508
K4D006	Protein TIC 214	0	5,503056	nucleotide binding	315
A0A3Q7JBH3	14_3_3 domain-containing protein	8,907435	0	signal transduction	401
A0A3Q7ILD3	5-methyltetrahydropteroyltriglutamate	5,278182	0	methionine biosynthetic process	765
A0A3Q7INF5	AAA+ ATPase domain-containing protein	8,688442	0	proteolysis	1,461
A0A3Q7HSF7	AAI domain-containing protein	7,066703	0	response to biotic stimulus	108
Q05540	Acidic 27 kDa endochitinase	13,382121	0	defense response to fungus	247
A0A3Q7IXY6	ADP/ATP translocase	10,660871	0	mitochondrial ADP transmembrane transport	386
A0A3Q7G528	Annexin	7,011662	0	response to stress	316
A0A3Q7EK65	Arginase	0	16,007418	putrescine biosynthetic process from arginine	664
A0A3Q7HEE3	Bulb-type lectin domain-containing protein	8,735564	0	Defense Signaling	297
A0A3Q7GY41	C3H1-type domain-containing protein	0	9,570218	Post-Transcriptional Regulation	1,942
Q5NE20	Carbonic anhydrase	12,059091	0	carbon utilization	321
Q9M513	Carboxypeptidase	3,84895	16,98765	proteolysis	498
A0A3Q7IHS3	Chitin-binding type-1 domain-containing protein	13,2994795	0	chitin catabolic process	329
P27522	Chlorophyll a-b binding protein 8, chloroplastic	16,955954	0	response to light stimulus	273
A0A3Q7IAX8	Chlorophyll a-b binding protein, chloroplastic	0	17,437874	photosynthesis	273
A0A3Q7JD76	Glyceraldehyde-3-phosphate dehydrogenase	0	16,14632	glucose metabolic process	450
A0A3Q7GDG7	Glyceraldehyde-3-phosphate dehydrogenase	13,472783	0	glucose metabolic process	338

A0A3Q7GB69	Glyceraldehyde-3-phosphate dehydrogenase	14,154289	0	glucose metabolic process	476
A0A3Q7EQ38	Glycinamide ribonucleotide synthetase	14,559219	3,6496935	chaperone cofactor-dependent protein refolding	1265
A0A3Q7HPN5	Histidine decarboxylase	5,1704764	0	organonitrogen compound biosynthetic process	1,631
A0A3Q7ITW6	Histone H2A	0	18,063513	DNA-binding	145
A0A3Q7J176	Histone H2A	17,597881	0	DNA binding	272
A0A3Q7IRR8	Isocitrate dehydrogenase (NADP(+))	6,963982	0	isocitrate metabolic process	520
A0A3Q7FKJ0	Leucine-rich repeat-containing N-terminal plant-type	15,807366	5,381088	defense response to other organism	464
A0A3Q7IIS3	Macrophage migration inhibitory factor homolog	10,950305	0	redox homeostasis	136
Q645N1	Malate dehydrogenase	0	14,481003	tricarboxylic acid cycle	346
A0A3Q7HGQ2	Malate dehydrogenase	16,319246	0	malate metabolic process	348
A0A3Q7I0C7	MFS domain-containing protein	7,769079	0	response to stimulus	572
A0A3Q7H3K1	MI domain-containing protein	0	10,603684	protein refolding	1,407
A0A3Q7FHW8	mitochondrial outer membrane protein porin of 34 kDa	0	7,2474008	Transmembrane Transport	338
A0A3Q7FLI5	Mitochondrial outer membrane protein porin of 34 kDa	13,08054	0	Transmembrane Transport	276
A0A3Q7G6R3	Pentacotripeptide-repeat region of PRORP	5,3117456	0	Regulation of Gene Expression	894
A0A3Q7IXE8	Peptidase_M28 domain-containing protein	11,039879	0	Protein Degradation and Peptide Metabolism	587
A0A3Q7EA02	Peptidyl-prolyl cis-trans isomerase	16,6429	5,4865446	protein folding	248
A0A3Q7FRK3	Peroxidase	5,5798907	16,583242	response to oxidative stress	721
A0A3Q7FNP2	PLAT domain-containing protein	5,287735	0	Lipid Transport and Signaling	193
K4CWC4	PR10 protein	15,936817	6,987407	response to biotic stimulus	160
I3QHF0	Proteinase inhibitor II	0	8,965204	protease inhibitor	148
I3QHE9	Proteinase inhibitor II	11,166905	0	protease inhibitor	148
A0A3Q7HG48	RING-type domain-containing protein	0	6,5115576	proteolysis	1,003

A0A3Q7J0I3	RUBISCO large subunit-binding protein subunit alpha	15,541964	6,574377	protein refolding	588
A0A3Q7HXW1	SCP domain-containing protein	16,10537	0	response to biotic stimulus	488
A0A494GA45	SCP domain-containing protein	16,757978	0	response to biotic stimulus	228
A0A3Q7G863	Serine hydroxymethyltransferase	10,918389	0	tetrahydrofolate metabolic process	517
A0A3Q7HUH7	SGNH hydrolase-type esterase domain-containing protein	0	4,9933414	Cell Wall Remodeling	244
A0A3Q7GBP8	Thioredoxin domain-containing protein	5,381353	0	regulation and signaling	296
A0A3Q7FZI5	Triosephosphate isomerase, cytosolic	7,4768577	0	glycolytic process	254
A0A3Q7GAU3	Uncharacterized protein	0	5,9536986	_	750
A0A3Q7IIS5	Vacuolar proton pump subunit B	13,132485	1,6840357	ATP metabolic process	489
A0A3Q7JQR2	Very-long-chain 3-oxoacyl-CoA synthase	0	6,1555476	fatty acid biosynthetic process	905
A0A3Q7FN07	V-type proton ATPase subunit C	4,6524267	0	Hydrogen ion transport	376
A0A3Q7J2P9	wound-induced proteinase inhibitor 1	16,325441	17,068975	negative regulation of peptidase activity	111
P05118	Wound-induced proteinase inhibitor 1	10,831639	0	response to wounding	111
Q8GT67	Xyloglucan-specific fungal	5,144642	0	proteolysis	438

Table S2. Identification of unique and differential proteins NT *versus* L12 BiP treatment inoculated. (NT): Non-transformed **plant; (L12 BiP)** Transgenic line.

Nº Acess	*Protein Name*	*Abundance L12 BiP*	*Abundance Non-transformed*	*Biologic Process*	*Aminoacids*
A0A3Q7HD09	23 kDa subunit of oxygen evolving system of photosystem II	0	17,65856	photosynthesis	258
A0A3Q7G3C1	23 kDa subunit of oxygen evolving system of photosystem II	0	5,399993	photosynthesis	710
A0A3Q7F9M7	50S ribosomal protein L12, chloroplastic	0	7,3996496	translation	290
K4CUW3	60S ribosomal protein L23	8,418745	0	translation	140
Q05540	Acidic 27 kDa endochitinase	9,254648	0	defense response to fungus	247
A0A3Q7IXY6	ADP/ATP translocase	11,763636	0	mitochondrial ADP transmembrane transport	386
A0A3Q7IP30	Alginate_lyase2 domain-containing protein	4,9342847	0	Polysaccharide Degradation	215
A0A3Q7JX44	ATP synthase delta chain, chloroplastic	0	5,3416386	proton motive force-driven ATP synthesis ATP	250
A0A0C5C9T7	ATP synthase epsilon chain, chloroplastic	0	12,279519	synthesis ATP	133
P60117	ATP synthase subunit alpha	0	15,197087	proton motive force-driven ATP synthesis	74
A0A0C5CEC7	ATP synthase subunit beta, chloroplastic	16,210062	17,332685	ATP binding	498
A0A3Q7IQN6	Avr9/Cf-9 rapidly elicited protein 75	0	9,4071	Defense Responses	141
A0A3Q7J601	beta-glucuronosyltransferase GlcAT14A-like	0	5,1921864	L-methionine salvage from methylthioadenosine	665
A0A3Q7IJL2	Bifunctional inhibitor/plant lipid transfer protein	16,677635	0	lipid transport	127
A0A3Q7J376	Bifunctional inhibitor/plant lipid transfer protein	0	6,90696	lipid transport	265
A0A3Q7HEE3	Bulb-type lectin domain-containing protein	5,040666	0	Defense Against Pathogens	297
A0A3Q7GSZ8	Calmodulin-binding domain-containing protein	0	5,07653	calcium-mediated intracellular signaling	1,303
Q5NE20	Carbonic anhydrase	0	12,278127	carbon utilization	321
A0A3Q7JD73	Catalase	8,515547	0	response to hydrogen peroxide	497
A0A3Q7IHS3	Chitin-binding type-1 domain-containing protein	11,344196	0	chitin catabolic process	329
P07370	Chlorophyll a-b binding protein 1B, chloroplastic	17,116463	0	photosynthesis, light harvesting in photosystem IS	265
A0A3Q7G195	Chlorophyll a-b binding protein, chloroplastic	0	18,139645	response to light stimulus	514
A0A3Q7I9X0	Chlorophyll a-b binding protein, chloroplastic	0	9,2936125	response to light stimulus	289
A0A3Q7GNU4	CP12 domain-containing protein	0	5,429525	negative regulation of reductive pentose-phosphate cycle	127
A0A3Q7H8G2	Cysteine proteinase 3	15,775349	17,14051	positive regulation of apoptotic signaling pathway	359
A0A3Q7H940	Cysteine proteinase 3	10,3277235	0	positive regulation of apoptotic signaling pathway	370
A0A3Q7F9X5	Cytochrome c domain-containing protein	5,002516	0	mitochondrial electron transport, cytochrome c to oxygen	112
A0A3Q7HRC2	DUF642 domain-containing protein	0	12,453285	Cell Wall Modification	371
A0A3Q7HX95	Fructose-bisphosphate aldolase	0	7,6677704	fructose 1,6-bisphosphate metabolic process/ glycolytic process	358

A0A3Q7EEZ8	Glucan endo-1,3-beta-D-glucosidase	10,866195	0	Plant defense	371
Q01412	Glucan endo-1,3-beta-glucosidase A	11,645434	0	defense response/carbohydrate metabolic process	336
A0A3Q7EHJ9	Glucose-1-Phosphate adenylyltransferase	0	5,337495	glycogen biosynthetic process	515
Q9ZR41	Glutaredoxin	0	16,571709	cellular response to oxidative stress	108
A0A3Q7JD76	Glyceraldehyde-3-Phosphate dehydrogenase	12,015998	0	glucose metabolic process	450
A0A3Q7GB69	Glyceraldehyde-3-Phosphate dehydrogenase	0	8,389529	glucose metabolic process	476
A0A3Q7GDG7	Glyceraldehyde-3-Phosphate dehydrogenase	0	5,2341156	glucose metabolic process	338
A0A3Q7H377	Glyco_18 domain-containing protein	9,098129	0	chitin catabolic process	376
A0A3Q7HZL1	LETM1 domain-containing protein	0	5,657101	intracellular monoatomic cation homeostasis	881
A0A3Q7I0C7	MFS domain-containing protein	0	7,440072	/response to stimulus	572
A0A3Q7GM40	NAD(P)-bd_dom domain-containing protein	0	8,52499	Energy Metabolism	496
Q9S8V0	Osmotin homolog (Fragment)	0	5,2829447	Osmotic Stress Response	27
P23322	Oxygen-evolving enhancer protein 1, chloroplastic	16,418581	17,640514	photosystem II assembly	329
P29795	Oxygen-evolving enhancer protein 2, chloroplastic	16,69885	0	calcium ion binding	258
P32045	Pathogenesis-related protein P2	12,100171	0	defense response to fungus	143
Q43143	Pectinesterase/pectinesterase inhibitor U1	5,0644093	0	cell wall modification	583
A0A3Q7FZH4	Pentacotripeptide-repeat region of PRORP domain-containing	5,7145166	0	RNA Processing and Maturation	792
A0A3Q7FSA0	Pentose-5-phosphate 3-epimerase	0	5,3995395	carbohydrate metabolic process	282
A0A3Q7IXE8	Peptidase_M28 domain-containing protein	9,226873	0	Protein Degradation and Peptide Metabolism	587
A0A3Q7G1J0	Peptidylprolyl isomerase	0	8,755832	Protein Folding	235
A0A3Q7FAE2	Peptidylprolyl isomerase	0	5,5392895	protein peptidyl-prolyl isomerization	221
A0A3Q7FRK3	Peroxidase	16,03974	6,983567	response to oxidative stress	721
A0A3Q7E7T2	Peroxidase	16,741158	0	response to oxidative stress	325
A0A3Q7GNU8	Peroxidase	15,696194	0	response to oxidative stress	322
A0A3Q7E8T9	Peroxidase	0	11,05181	response to oxidative stress	326
A0A3Q7HGJ9	Phosphoglycerate kinase	8,364711	16,227102	gluconeogenesis	883
A0A3Q7JFN4	Photosystem II 5 kDa protein, chloroplastic	0	16,318045	photosynthesis	113
Q672Q6	Photosystem II oxygen-evolving complex protein 3	15,295007	16,742435	photosynthetic electron transport chain	230

A0A3Q7HFK3	photosystem II repair protein PSB27-H1, chloroplastic	0	7,0672183	photosystem II assembly	197
A0A3Q7FFY2	Photosystem II stability/assembly factor	3,4107676	12,994947	photosynthesis	387
Q0ZPA3	Plastid lipid associated protein CHRC	16,10505	17,812954	Lipid Metabolism	326
P17340	Plastocyanin, chloroplastic	17,002916	18,331306	Electron Transport in Photosynthesis	170
A0A3Q7FNP2	PLAT domain-containing protein	8,443889	0	Stress response	193
B2LW68	PR1 protein	7,2294254	0	response to biotic stimulus	179
K4CWC4	PR10 protein	15,451811	0	response to biotic stimulus	160
Q672Q7	Protein maintenance of psii under high light 1	0	5,4491215	regulation of protein complex stability	238
A0A3Q7IM48	Protein-serine/threonine phosphatase	8,450963	0	peptidyl-threonine dephosphorylation	615
A0A3Q7IED7	PSI-K	0	5,167059	photosynthesis	130
A0A3Q7FZ81	Reverse transcriptase Ty1	12,576017	0	Regulation of Gene Expression	645
A0A3Q7ENE0	Ribose-5-phosphate isomerase	0	5,6335564	pentose-phosphate shunt, non-oxidative branch	293
A0A3Q7I3Q3	Ribosome-recycling factor, chloroplastic	0	5,612397	plastid translation	267
A0A3Q7IPE9	RIX1 domain-containing protein	5,3882623	0	rRNA processing	852
A0A3Q7H4L7	Sucrose-phosphate synthase	5,189914	0	sucrose biosynthetic process	1,054
A0A3Q7GUW9	Superoxide dismutase	15,728191	16,754358	response to oxidative stress	278
A0A3Q7H4P9	Thioredoxin domain-containing protein	5,363535	16,151642	zinc ion binding	1,388
A0A3Q7GZ46	Thioredoxin domain-containing protein	0	12,6966	response to oxidative stress	178
A0A3Q7E9H0	Thioredoxin-dependent peroxiredoxin	11,292919	0	response to oxidative stress	267
A0A3Q7EN13	TPM_phosphatase domain-containing protein	0	10,90633	Carbohydrate Metabolism	289
P05118	Wound-induced proteinase inhibitor 1	0	7,11679	response to wounding	111

Table S3. Identification of gargal Proteins NT *Versus* L12 BiP Treatment Non- inoculated. (NT): Non-transformed **plant; (L12 BiP)** Transgenic line.

Gargal Proteins (NT versus L12 BiP) Non- Inoculated (Mean > 2410.40)							
Protein access	***Value***	***Protein access***	***Value***	***Protein access***	***Value***	***Protein access***	***Value***
A0A3Q7GWM7	2485,315175	A0A3Q7HRF8	3724,672642	A0A3Q7IMS6	6489,433932	A0A3Q7IZZ5	15155,4352
A0A3Q7J9M4	2485,315175	A0A3Q7FW96	3823,88788	A0A3Q7IBX7	6624,03438	A0A3Q7HQA3	16366,10216
A0A3Q7FPI2	2612,355548	A0A3Q7I9E4	3827,476636	TAL	6792,080219	A0A3Q7F8L3	17475,00494
A0A3Q7IAQ0	2612,355548	A0A3Q7IM60	4362,294941	A0A3Q7EHA9	6872,435819	A0A3Q7IS42	19918,71526
A0A3Q7F5F8	2655,137079	A0A3Q7H0S8	4431,599749	A0A3Q7HFR4	6872,435819	A0A3Q7HS81	20667,46445
A0A3Q7IDI1	2655,137079	A0A3Q7GAD2	4542,926516	A0A3Q7J4V1	6872,435819	2-CP1	22160,09331
A0A3Q7H7H6	2691,316301	A0A3Q7FGC1	4761,513778	HSP70	6925,538637	2-CP2	22160,09331
A0A3Q7EP38	2739,006253	A0A3Q7IT88	4761,513778	PURA	7082,910382	EIF(iso)4G	22582,21165
A0A3Q7E8I1	2749,553918	ASS	4861,26403	PURA-2	7082,910382	A0A3Q7EJA6	24894,37948
A0A3Q7F897	2749,553918	HDR	4862,649243	A0A3Q7EVV1	7270,875868	A0A3Q7H3D4	25988,84886
A0A3Q7F529	2781,210675	A0A3Q7IZ93	4958,10719	A0A3Q7HEZ3	7270,875868	VATL_SOLLC	29776,35516
A0A3Q7GNY6	2792,879308	A0A3Q7GGR3	5056,234257	A0A3Q7IDX8	7270,875868	ARG2	33925,54846
A0A3Q7I742	2792,879308	A0A3Q7FAC9	5126,64476	ca2	7368,810944	A0A3Q7EIY6	34082,34518
A0A3Q7J221	2880,61171	A0A3Q7FVP3	5126,64476	A0A3Q7E824	8180,125815	A0A3Q7GN20	34082,34518
A0A3Q7GLC6	2883,605843	A0A3Q7GD18	5126,64476	A0A3Q7G087	8211,257723	A0A3Q7JX44	34082,34518
A0A3Q7IQV2	2883,605843	A0A3Q7E9H1	5200,193687	A0A3Q7HXS8	8541,333415	ndhK	34142,36614
A0A3Q7EMK5	2987,557531	ER69	5200,193687	A0A3Q7G242	8899,965674	A0A3Q7EM39	35039,77545
A0A3Q7HD29	3157,985294	A0A3Q7JJ90	5201,228754	A0A3Q7IKJ5	9366,972382	A0A3Q7EM41	35039,77545
G8Z261_SOLLC	3190,60234	A0A3Q7G7N7	5371,603901	CYN	9741,625	A0A3Q7EUT1	35732,81952
A0A3Q7HNA9	3233,963412	A0A3Q7IKC4	5572,545373	A0A3Q7I365	9832,324319	A0A3Q7JBD6	36478,7452
LeSSADH	3270,298978	A0A3Q7JLM2	6057,890976	A0A3Q7J1M0	10303,41193	psbB	36523,10817
A0A3Q7H9K1	3327,990495	A0A3Q7FDY7	6073,87177	A0A3Q7FPD9	10810	GPI	40473,11028
psaC	3328,7944	A0A3Q7HT00	6073,87177	A0A3Q7IMY6	10815,19643	A0A3Q7HGN6	43618,30027
A0A3Q7HB86	3349,240571	A0A3Q7J9P7	6073,87177	A0A3Q7FCE4	11586,46177	A0A3Q7J643	43618,30027
A0A3Q7I518	3349,240571	A0A3Q7EQD7	6132,025657	A0A3Q7G863	11586,46177	A0A3Q7J647	43618,30027

A0A3Q7I4P2	3390,086882	A0A3Q7GNF6	6132,025657	atpH	12775,27604	A0A3Q7I041	51061,93972
A0A3Q7EHP3	3473,731602	A0A3Q7HN29	6132,025657	A0A3Q7EU87	13153,59603	A0A3Q7FZZ0	58481,95373
TPx1	3473,731602	A0A3Q7JDH2	6132,025657	A0A3Q7FZI5	13153,59603	A0A3Q7GUK7	58481,95373
A0A3Q7ILP6	3476,398268	A0A3Q7JE99	6132,025657	A0A3Q7GRE0	13153,59603	A0A3Q7IIS5	68391,53115
A0A3Q7GZP7	3478,585402	A0A3Q7J033	6136,684466	A0A3Q7IHG0	13153,59603	A0A3Q7EK01	107523,2261
A0A3Q7HRK7	3478,585402	A0A3Q7I5I0	6251,432978	ldh	13428,74457	A0A3Q7JN10	133745,4936
A0A3Q7FRU0	3525,868512	A0A3Q7I6C0	6251,432978	AS	14078,57938	A0A3Q7EUX7	136659,9259
A0A3Q7EQJ0	3614,259466	A0A3Q7JAZ3	6431,380751	ndhC	14250,10882	A0A3Q7I6P6	155924,0294
A0A494G8L6	3670,680249	Ca3	6470,488641	A0A3Q7GLM4	14755,72544	A0A3Q7EQ38	297734,5699
A0A3Q7G2Q9	3724,672642	ca1	6470,488641	A0A3Q7G9W0	14934,89768	A0A3Q7G5K0	303857,6066

Table S4. Identification of gargal Proteins NT *Versus* L12 BiP Treatment Inoculated with *M. perniciosa*. (NT): Non-transformed plant; **(L12 BiP)** transgenic line**.**

Gargal Proteins (NT versus L12 BiP) Inoculated (Mean > 3232.35)											
Protein access	***Value***	***Protein access***	***Value***	***Protein access***	***Value***	***Protein access***	***Value***	***Protein access***	***Value***	***Protein access***	***Value***
SUS3	3315,9156	A0A3Q7G4Q1	4766,8919	A0A3Q7JG72	6734,7103	A0A3Q7JFK3	10123,957	A0A3Q7G9T7	15106,341	A0A3Q7GYB4	28715,302
A0A3Q7J3I1	3315,9156	PAD1-2	4939,1044	rps14-2	6771,5807	A0A3Q7GME0	10123,957	ndhJ	15545,498	A0A3Q7FRC7	29436,992
A0A3Q7I629	3315,9156	PAD1	4939,1044	psbD	6797,2847	A0A3Q7EFX0	10123,957	psbK	15642,131	atpH	31358,968
A0A3Q7FNU1	3315,9156	K4BB06_SOLLC	4939,1044	A0A3Q7IZ93	6840,9898	ndhK	10191,983	A0A3Q7HR13	15919,801	A0A3Q7GRY8	32810,48
A0A3Q7F670	3315,9156	A0A3Q7G580	4939,1044	A0A3Q7G7F0	6849,3918	A0A3Q7G5K0	10340,856	A0A3Q7HS19	16002,957	K4B4Z0_SOLLC	32911,185
A0A3Q7EAM0	3324,1683	A0A3Q7FIL8	4939,1044	A0A3Q7JBJ2	6935,7852	A0A3Q7FPC0	10614,276	TAL	16109,171	A0A3Q7G6Z4	35712,147
A0A3Q7EDG4	3341,1638	A0A3Q7FEV4	4939,1044	Acx1B	7010,2555	A0A3Q7FM90	10614,276	A0A3Q7G087	17298,5	GPI	36818,745
SlFBA7	3398,1971	A0A3Q7GNB3	4939,1375	ndhI	7122,5093	A0A3Q7H226	10614,298	SSTLE1	17534,143	A0A3Q7J2W8	37016,827
A0A3Q7GCG9	3421,827	A0A3Q7FL83	4939,1375	ndhE	7148,0869	psbL	10825,648	rps16	17990,469	A0A3Q7H2A5	38321,481
A0A3Q7FSA0	3421,827	A0A3Q7J3Q6	4939,1536	A0A3Q7GAY7	7254,414	A0A3Q7HY56	11257,787	K4CG62_SOLLC	18305,327	A0A3Q7JPK6	38321,481
A0A3Q7GGP4	3463,8569	A0A3Q7IZW2	4939,1536	atpI	7407,4241	ER49	11313,904	A0A3Q7EQN8	18930,916	psbB	44402,987
A0A3Q7FAU0	3463,8569	A0A3Q7I6A1	4939,1536	A0A3Q7FPK3	7420,2516	psbJ	11412,006	ca2	19442,131	A0A494G9Y0	44891,896
A0A3Q7I9A3	3642,1171	A0A3Q7GA27	4939,1536	PME2.1	7583,3327	K4B017_SOLLC	12381,876	A0A3Q7FYD2	19650,945	A0A3Q7J2E6	45154,285
A0A3Q7ECP4	3657,6796	A0A3Q7FYL2	4939,1536	PME1.9	7583,3327	A0A3Q7H9K1	12469,75	A0A3Q7GG28	19749,809	rps12-A	53447,604
A0A3Q7JDG0	3671,9374	A0A3Q7F6F6	4939,1536	A0A3Q7HFT8	7583,3327	AgpL3	12512,925	A0A3Q7FFN7	20601,6	A0A3Q7EQJ0	58987,548
A0A3Q7EMS1	3758,6163	H6WYS2_SOLLC	4944,5276	psbM	7855,8455	AgpL1	12512,925	A0A3Q7F8T6	20601,6	A0A3Q7JD72	59686,647
A0A3Q7I001	3763,9117	A0A3Q7G5P1	5084,2201	A0A3Q7IZG8	8097,0259	AGP-S2	12512,925	A0A3Q7JD24	20601,6	A0A3Q7HGJ9	60877,83
psaC	3797,9598	A0A494G8L6	5181,2653	H9BYP6_SOLLC	8119,7485	A0A3Q7JX44	12832,549	A0A3Q7GC33	20601,6	A0A3Q7HWQ3	64051,313
atpF	3831,9567	A0A3Q7IHG0	5235,1625	A0A3Q7IXC3	8119,7485	A0A3Q7GN20	12832,549	A0A3Q7G4Q5	20832,1	A0A3Q7EA02	65894,534
HXK4	3911,9456	A0A3Q7GRE0	5235,1625	psbE	8134,6654	A0A3Q7H7H6	12850,381	A0A3Q7HXS8	20914,131	A0A3Q7FHW2	68203,928
A0A3Q7I392	4014,3365	A0A3Q7FZI5	5235,1625	rpl36	8161,2362	A0A3Q7J4G5	12904,842	A0A3Q7G242	20993,578	A0A3Q7F545	68203,928
A0A3Q7I6S9	4044,7545	A0A3Q7EU87	5235,1625	A0A3Q7IKC4	8345,1053	A0A3Q7HGI8	12904,842	A0A3Q7J1M0	21194,241	A0A3Q7EXD9	68203,928
A0A3Q7I5P6	4044,7545	A0A3Q7GMQ4	5386,8223	A0A3Q7JLM2	8601,2695	A0A3Q7I756	13022,095	A0A3Q7EIY6	21265,777	A0A3Q7FPD9	72784,642
A0A3Q7GAX9	4098,2905	A0A3Q7FUI9	5386,8223	A0A3Q7J9P7	8696,0201	A0A3Q7GJA8	13022,095	A0A3Q7I9E4	23172,346	A0A3Q7EHC5	77451,507
A0A3Q7I0X4	4104,5555	NR	5423,4325	A0A3Q7HT00	8696,0201	A0A3Q7FY19	13022,095	ndhC	23660,346	rps16-2	77919,139

K4BQD9_SOLLC	4122,0147	NIA	5423,4325	A0A3Q7FDY7	8696,0201	psbA	13032,796	A0A3Q7J882	24353,836	2-CP2	80095,125
BCCP1	4203,2248	A0A3Q7IKJ5	5629,0236	A0A3Q7G214	8731,4742	CYN	13179,625	PIIF	24810	2-CP1	80095,125
A0A3Q7HD15	4248,7164	CAB12	5669,8877	A0A3Q7IZZ5	8818,9709	A0A3Q7F0C1	13180,218	A0A3Q7IJQ0	24859,193	A0A3Q7JBD6	84899,284
A0A3Q7HAH2	4248,7164	CAB11	5669,8877	A0A3Q7EJ55	9025,6825	Cyp-3	13209,5	A0A3Q7IB41	24933,214	A0A3Q7IWJ3	101181,92
A0A3Q7EQJ7	4248,7164	rpoA	5821,2115	ca1	9229,6395	A0A3Q7FPW8	13319,707	A0A3Q7J415	25604,833	A0A3Q7F9X5	101915,2
psbF	4251,2745	CAT2-2	5876,0357	Ca3	9229,6395	A0A3Q7F6F2	14016,95	rps18	26191,72	A0A3Q7FFY2	102840,97
atpB	4344,6625	A0A3Q7JD91	6149,4094	A0A3Q7JAZ3	9712,3285	A0A3Q7EMX2	14016,95	A0A3Q7J2T1	26192,999	CYC_SOLLC	103120,53
A0A3Q7JPG0	4409,2265	A0A3Q7I365	6208,6038	A0A3Q7E9H4	9720,3186	Atg6	14633,44	A0A3Q7HBK4	26192,999	ldh	103136,39
SlALDH11A3a	4448,7139	psbH	6235,3949	A0A3Q7H274	9945,7021	A0A3Q7IMY6	14635,196	A0A3Q7IKB1	27142,307	PMEU1	103595,02
psbC	4462,021	A0A3Q7I9T7	6311,7747	A0A3Q7GMM2	9945,7021	RPL8	14859,649	A0A3Q7H5X9	27820,701	A0A3Q7FSD1	118236,54
A0A3Q7H0L5	4561,1113	A0A3Q7J4W6	6338,899	A0A3Q7IDX8	10000,631	A0A3Q7J4V1	14996,829	A0A3Q7F8L3	28258,577	rbcL	129393,85
LAT56	4599,2809	CAB7-2	6667,9169	A0A3Q7HEZ3	10000,631	A0A3Q7HFR4	14996,829	A0A3Q7I6B5	28715,302	A0A3Q7HJ28	135258,12
A0A3Q7HWE4	4677,4094	CAB7	6667,9169	A0A3Q7EVV1	10000,631	A0A3Q7EHA9	14996,829	A0A3Q7HJU1	28715,302	SO	217074,42
